# Towards Intelligent Pain Monitoring Systems: A Survey of Recent Technologies and Methods

**DOI:** 10.3390/s26082447

**Published:** 2026-04-16

**Authors:** Atif Naseer, Nahla Tayyib, Sidra Rashid

**Affiliations:** 1 Deanship of Postgraduate Studies and Research, Umm Al-Qura University, Makkah 21955, Saudi Arabia; 2 Clinical Nursing Practices, Faculty of Nursing, Umm Al-Qura University, Makkah 21955, Saudi Arabia; natayyib@uqu.edu.sa; 3 Department of Computer Sciences, Quaid-i-Azam University, Islamabad 45320, Pakistan; nssidra.rashid@gmail.com

**Keywords:** pain, automatic pain monitoring, Internet of Things (IoT), computer-vision, multimodal

## Abstract

Pain is a profoundly stressful experience that significantly impacts an individual’s daily life. In many situations, people can express the intensity of pain via some observable physical actions like crying or shouting. However, in cases where the patient is non-communicative, they cannot convey their feelings through these actions. In both scenarios, automatically monitoring pain intensity using technology presents a considerable challenge. In the literature, researchers have presented numerous techniques for automatic pain monitoring using multiple approaches. This technological survey paper aims to provide an overview of current advancements in the field of automatic pain monitoring. In this paper, we present a taxonomy that summarizes our survey on the utilization of technology areas for monitoring pain automatically. Those technologies are based on Internet of Things (IoT), computer vision, and multimodal techniques. These technologies utilize various modalities, including physiological signals, facial expressions, vocalizations, and behavioral patterns, to detect and quantify pain. The paper discusses the advantages and limitations of each modality, as well as the challenges faced in developing accurate and reliable pain monitoring systems. Additionally, the paper surveys the current state of research in this field, including the development of machine learning algorithms and wearable devices for pain monitoring. Overall, this paper provides a comprehensive overview of the current state of automatic pain monitoring technology and highlights areas for future research and development. This paper also creates a keyword map that will serve as a valuable resource for researchers, enabling them to refine their investigations by identifying frequently used terms and emerging trends within each domain.

## 1. Introduction

Pain is a profoundly distressing sensation for any individual, significantly impacting their daily life. According to the International Association for the Study of Pain (IASP), pain is defined as “an unpleasant sensory and emotional experience associated with actual or potential tissue damage or described in terms of such damage” [1]. Pain perception (phantom pain syndrome) may occur due to neurological processes even in the absence of direct physical stimuli. This addition helps emphasize the complexity of pain assessment and further supports the need for advanced monitoring techniques. In many situations, people can express the intensity of pain via some physical action like crying or shouting. In some situations, the patient cannot express the feeling through these actions. In both cases, it is very difficult to monitor the intensity of pain automatically using some technology. In the literature, researchers presented many techniques to monitor the pain automatically using multiple techniques. The major effects of pain intensity involve mental health [2], sleep reduction [3], loss of appetite [4], anxiety, and depression [5]. Therefore, pain monitoring is very important to detect as early as possible to reduce the intensity of these problems. Pain can have a significant impact on human health, both physically and mentally. Chronic pain can lead to reduced mobility and activity, which can contribute to weight gain, muscle weakness, and other health issues. Additionally, chronic pain can interfere with sleep, which can further exacerbate these negative effects on health. Effective pain management is important for maintaining overall health and well-being. The pain threshold is the minimum level of irritation of the nervous system at which a person begins to feel pain, which is strictly individual, and also strongly depends on the psychological state of the person (for example, under severe stress) and may interfere with the normal feeling of pain. Pain intensity is a subjective experience that can be difficult to measure objectively. The self-reported pain is easy to identify, and its intensity can be monitored by communicating with the patient. However, there are several commonly used scales for describing pain intensity. Here are some of the most common types of pain intensity scales:

1. Verbal Rating Scale (VRS) [6]: In VRS, adjectives are used to describe different levels of pain. The adjectives include “no pain,” “mild,” “moderate,” “severe,” and “worst pain” to measure pain intensity. It is often used for adults who can clearly articulate their pain levels.
2. Visual Analog Scale (VAS) [7]: It consists of a horizontal line with numbers ranging from 0 to 10. The patient identifies a point on the line to represent their pain. It is effective in measuring minute pain changes.
3. Numerical Rating Scale (NRS) [8]: Similar to the VRS, this scale asks the patient to rate their pain on a scale of 0 to 10, with 0 representing no pain and 10 representing the worst pain imaginable. This scale is widely applicable across various patient populations due to its simplicity.
4. Faces Pain Scale-Revised (FPS-R) [9]: This scale can be used for children or an adult experiencing difficulties in using a verbal or numerical scale. It uses a series of faces with varying expressions, and the patient is asked to choose the face that best represents their pain. It is ideal for pediatric patients or individuals with cognitive impairments.
5. Wong–Baker FACES Pain Rating Scale [10]: Similar to the FPS-R, this scale also uses a series of faces with varying expressions, and the patient is asked to choose the face that best represents their pain. This scale is also commonly employed in pediatric settings and for patients with communication barriers.

Different types of pain intensity scales may be more appropriate for different patients and situations, and healthcare providers may use multiple scales to get a more comprehensive understanding of a patient’s pain experience. In major cases, the patients explain their pain (this self-reported pain is easy to identify), and its intensity can be monitored by communicating to the patient. In medical sciences, numerous methodologies exist to measure the intensity of pain with self-reported patients. Some of the commonly used methods are the Visual Analog Scale for Pain (VAS) [7], Numeric Rating Scale (NRS) [8], pain diaries [11], and verbal descriptions [12]. In other cases when the patients are non-communicative due to critical illness, they are not able to report the pain, and their pain intensity cannot be measured by ordinary means. These patients are monitored by the medical staff and there is no means to intensify the pain measures. The literature also mentioned some observational methods like the Behavioral Pain Scale (BPS) [13], Pain Assessment in Advanced Dementia (PAINAD) [14], Neonatal Infant Pain Scale (NIPS) [15], or Prkachin–Solomon Pain Intensity (PSPI) scale [16] to monitor the pain of these non-communicative patients.

Automated pain recognition refers to the use of technology, such as machine learning algorithms, to automatically detect and quantify pain based on physiological, behavioral, or facial features [17]. This technology has the potential to improve pain assessment and management in clinical settings, particularly for patients who have difficulty communicating their pain or whose pain is not visible. Automated pain recognition systems have been developed using a variety of data sources, including physiological signals such as heart rate and skin conductance, behavioral cues such as movement or vocalizations, and facial expressions. While automated pain recognition is still an emerging field, it has the potential to enhance pain management and pain assessments within a number of clinical scenarios. However, there are also concerns about the reliability and accuracy of these systems, as well as potential ethical concerns related to privacy and data security. Automated pain recognition offers several key advantages: it can be considered as a diagnostic tool for detecting complications, it benefits clinical staff and inpatients during night stays by providing continuous monitoring, it helps overcome language barriers, and it can validate self-reporting scales. These benefits underscore its potential to revolutionize pain management.

Automatic pain monitoring is very challenging task. The wrong measurement of pain will lead to some serious consequences for patients. Various technologies are currently used by researchers that help in automatic pain monitoring. Facial expressions and sensors on the body are the two major inputs that help in detecting the pain intensity of the patients [18]. Facial expressions are one of the major indicators of pain detection, as in most cases, facial expression changes with pain intensity. The researchers used these expressions to automate the pain monitoring mechanism using computer vision or sensor-based techniques. Many researchers use wearable devices or body motion sensors to monitor patient activities and body movement. Brain signals can also be one of the factors to monitor pain intensity. EMG, EGG, and skin sensors could be another way that can help with pain monitoring.

In this work, we present a taxonomy to classify the existing work of automatic pain monitoring. The taxonomy is divided into three main technologies namely Internet of Things (IoT), computer vision, and multimodal. The aim of this paper is to structure the progress of these technologies over the last decade. This paper will cover the technology used in automatic pain detection, the dataset used, the features extracted from the approach, and the impact of the research on the patient. The taxonomy will help the researchers to categorize the work according to the technology. We classified the work based on the classification of pain. This work categorizes the public datasets utilized in automatic pain monitoring and conducts a comparative analysis of them. This work also created a keyword map that will help the researchers to refine their research based on the frequently used keywords in each domain.

The remainder of this paper is structured as follows. Section 2 discusses the methodology and evaluation of the research. Section 3 is about the classification of pain. It describes the types of pain according to their category that will help the technology to estimate the pain. Section 4 presents the taxonomy used in this paper. Section 5 is about the publicly available datasets for automatic pain monitoring. Section 6 presents the limitations, challenges, and future directions. Section 7 concludes the paper.

## 2. Research Methodology and Initial Evaluation

This survey systematically reviews studies related to pain monitoring systems. We explore pain monitoring methods across different modalities. We have searched on academic research platforms including ACM Digital Library, IEEE Xplore, Elsevier, Google Scholar, PubMed Central, and SpringerLink. We include relevant publications up to 2025. Two categories of keywords were used in the literature search. The first set, “pain monitoring”, “automatic pain monitoring”, “intelligent pain monitoring”, and “pain monitoring methods”, was used to define the overall scope of the research. The second set focused on modality-specific approaches to pain assessment, including “computer vision–based pain monitoring”, “facial expression–based pain monitoring”, “speech–based pain monitoring”, “iris tracking–based pain monitoring”, “multimodal pain monitoring”, “IoT-based pain monitoring”, “wireless pain monitoring”, “body sensor–based pain monitoring”, and “pain classification”.

At the beginning of the review process, an initial pool of 135 research articles was selected based on relevance to the broad domain of pain monitoring. Following a thorough screening process involving abstract and full text evaluation, eighty publications were identified as directly related to the scope of our survey. Over these years, pain monitoring methods have made substantial progress in terms of the modalities and robustness of pain assessment systems.

## 3. Classification of Pain

**Figure 1:**
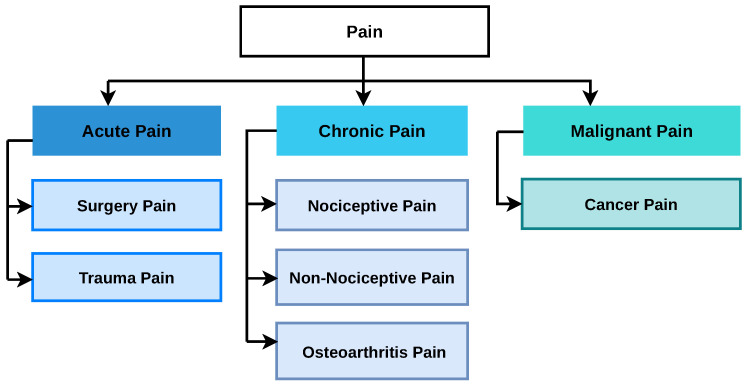
Classification of pain.

Pain occurs when an uncomfortable or unpleasant event occurs to the body. It can be caused by an accident or if something hurts. While broadly categorized, it is important to note that pain experiences can be complex, often overlapping between categories. Pain is classified into three groups majorly [19,20]. The first type of pain is called acute pain that can be caused by any surgery, broken bones, or any dental work. Chronic pain on the other hand is a continuous type of pain that comes and goes with time. Chronic pain is linked to some body conditions like headache, nerve pain, back pain, or fibromyalgia and arthritis. Another type of pain that is very close to chronic pain is malignant pain that can be categorized as cancer pain. Figure 1 shows the classification of pain in detail where these three major classifications of pain are further divided into subcategories.

### 3.1. Acute Pain

Acute pain refers to pain that comes suddenly and lasts for a limited time (less than 3 months) [21]. The damage of tissues, e.g., organs, muscles, and bones, may cause acute pain. Acute pain happens with emotional issues and anxiety often.

#### 3.1.1. Surgery Pain

Surgery pain comes after any surgery procedure. It is a normal part of healing, and it varies from person to person. This pain can range from mild discomfort after minor procedures to severe pain following major operations, but its cause is always identifiable and directly linked to surgical intervention. Every time, we know the cause of the pain.

#### 3.1.2. Trauma Pain

Trauma pain is usually severe pains that is caused from actual or potential tissue damage during trauma [22]. This can include physical injuries such as fractures, deep cuts, or muscle bruises, and sometimes psychological factors can also contribute to the overall pain experience. It can be physical fractures, muscle bruises, or sometime psychological factors.

### 3.2. Chronic Pain

The International Association for the Study of Pain (IASP) defines chronic pain as pain that lasts or recurs for longer than 3 months [23]. It can resist medical treatment generally. It comes with long-term illness, e.g., osteoarthritis. In some cases, such as with fibromyalgia, pain is one of the main traits of the condition. Chronic pain may result from nerve damage, tissue injury, or degeneration of joint cartilage. Nerve damage happens very often.

#### 3.2.1. Nociceptive Pain

The IASP defines nociceptive pain as “pain that arises from actual or threatened damage to non-neural tissue and is due to the activation of nociceptors” [19]. It describes a normal physiological response to tissue damage resulting from inflammatory processes, trauma, or a non-healing injury. There are two categories of nociceptive pain: somatic pain and visceral pain. Somatic pain refers to the musculoskeletal system, e.g., bone fracture and stomach. Visceral pain is felt indirectly, e.g., internal organ injury.

#### 3.2.2. Non-Nociceptive Pain (Neuropathic)

Neuropathic pain is caused by disease or a lesion in the somatosensory system [24]. It results from abnormal neural activity. Unlike nociceptive pain, it does not arise from tissue damage but from damage to the nervous system itself. It may be peripheral or central based on the location of the lesion in the nervous system. Examples include diabetic neuropathy, sciatica, or post-herpetic neuralgia, often described as burning, shooting, or tingling sensations.

#### 3.2.3. Osteoarthritis Pain

Osteoarthritis pain on the other hand is caused by joint degeneration, obesity, and sometimes other conditions like rheumatoid arthritis [25].

### 3.3. Malignant Pain

Malignant is a term used to describe cancer and cancer-related conditions [26]. Malignant cells invade nearby tissues in an uncontrolled manner. They spread through the lymph system and blood to other parts of the body.

#### Cancer Pain

This pain is caused by cancer or cancer treatments [21]. It can be classified as acute or chronic and can be caused by a variety of factors, such as tumor growth, nerve damage, or chemotherapy.

## 4. Taxonomy

**Figure 2:**
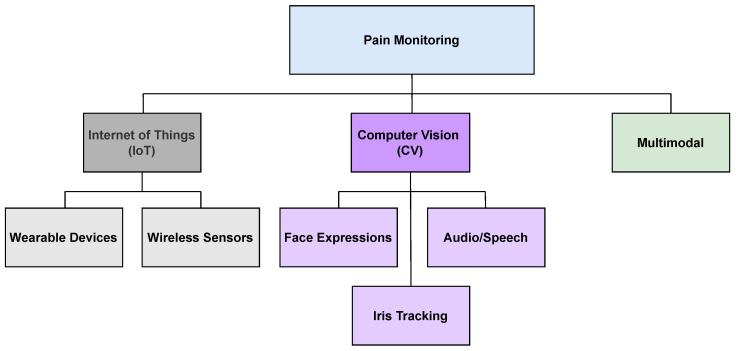
Taxonomy for classification of automatic pain monitoring techniques.

Figure 2 summarizes our current taxonomy that classifies the various technologies used in automatic pain management. This taxonomy was developed through a comprehensive review of the literature, categorizing approaches based on their primary technological foundation and data acquisition methods. These technologies are used by many researchers to automatically detect the pain and measure its severity. The taxonomy classifies the technologies used in automatic pain management into Internet of Things (IoT), computer vision (CV), and multimodal. Further, these technologies are categorized into their sub-areas for further studying. While this classification aims for comprehensiveness, it is acknowledged that some emerging or hybrid approaches might exhibit characteristics that span multiple categories, indicating the dynamic nature of this field of research.

### 4.1. Computer Vision Approaches

#### 4.1.1. Facial Expression Based Approaches

Computer vision approaches for automated pain detection face inherent challenges, including variations in facial expressions due to individual differences, cultural backgrounds, and the influence of external factors like lighting conditions on image quality. In health informatics and medical diagnostics, automated pain detection is highly desirable. The analysis of facial images is performed using machine learning algorithms. It is a challenging approach for pain detection and intensity analysis in the health domain. The research community has developed many advanced techniques to analyze facial expressions and predict the presence of pain. It is an important part of diagnostics for patients who are unable to speak or describe pain intensity. Table 1 shows the literature available for 2012–2023 for automated pain detection through facial expressions. It includes the dataset, preprocessing, processing (feature extraction and modeling technique), validation (stimuli, subject), and performance metrics for each paper.

In early efforts, mostly Gabor filter and principal component analysis (PCA) were used to improve the performance of pain detection algorithms. An automated pain detection framework was designed by the extraction of features from facial images [27]. The author used PCA and conducted experiments at both frame and image levels using the UNBC-McMaster Shoulder Pain Expression Archive. There is a variety of features explored by authors for pain monitoring. Kaltwang et al. [28] proposed appearance features and landmarks to analyze facial images. It includes Discrete Cosine Transformation (DCT), Relevance Vector Regression (RVR), and Local Binary Pattern (LBP). The fusion of these features leads to better estimation of pain levels as compared to particular feature-based pain estimation. The Prkachin and Solomon Pain Intensity (PSPI) metric is frequently used for pain. It was developed to classify four levels of pain [29]. To detect the pain level, support vector machines (SVMs) are separately trained on a frame-by-frame level. Experiments are performed on the UNBC-McMaster Pain database. Another author used multiple kernel SVMs for pain detection [30] from spatial and spatial–temporal features. This method uses feature fusion for pain detection and multi-task fusion for pain location detection. The experiments on the UNBC-McMaster Shoulder Pain database show better performance as compared to other methods for pain detection. Emerging machine learning techniques have great potential to diagnose certain medical conditions. Out of these techniques, deep neural networks outperform in feature identification from facial images to monitor pain. They present a comparative analysis of the Off-the-Shell CNN architectures, including MobileNet, GoogleNet, ResNet-50, ResNet18, and DenseNe-t161 [31]. These networks are deployed in standalone or feature extractor mode. Their outputs were employed to make the classification for the UNBC-McMaster Shoulder Pain dataset using Support Vector Regression (SVR) and Random Forest Regression (RFR). The obtained results highlight the usefulness of the hidden CNN layers for automatic pain estimation. Another approach [32] uses CNN for facial features learning; these features are linked with long short-term memory to achieve a temporal relationship between video frames. In [33], the author presents facial expressions as binary codes; these binary codes help in classifying the difference in pain intensity levels. The face video is divided into non-overlapping equal-length segments. Features are extracted using CNN. Deep learning-based pain classification from facial images data is proposed by Bargshady et al. [34]. Two different RNN models were used; they were pre-trained with a VGG Face CNN model and were later combined as a single network to estimate the intensity level of pain. Bargshady et al. [35], in his enhanced work, developed a deep neural network-based algorithm to detect four different pain levels.

Multidimensional convolution neural networks are also frequently explored in the literature due to their good performance. Huang et al. [36] have also proposed an end-to-end hybrid network for image multidimensional feature extraction. They designed a 3D-CNN to capture the spatiotemporal features, 2D-CNN to capture the spatial features, and 1D-CNN to capture geometric information from facial landmarks. These three features are fused and fed to the regression model. Similarly, 3D deep model for dynamic spatiotemporal representation of faces in video sequences is discussed in [37]. Cross-Architecture Knowledge Transfer is discussed as an effective method in training 3D CNN using pre-trained 2D architecture. The UNBC-McMaster Shoulder Pain and BioVid databases are used for evaluation. In [38], a self-supervised learning system is presented for pain assessment. In [39], a Multimodal Intensity Pain (MIntPAIN) database is introduced for pain level recognition. Their model recognizes five pain levels using independent visual modalities, CNN, and LSTM models. For automated emotion recognition and pain detection, the dynamics of facial expressions are represented in [40]. Several appearance-based descriptors are generated at each frame from a temporal sequence of facial photographs. The dynamics of facial expressions concerning the chosen feature–scale pair are represented by the Hankel matrix constructed from each time series. A boosting strategy is utilized to train a powerful classifier using the set of Hankel matrices created by altering the feature type and scale. Feature and scale selection during training is done via a random subspace projection. The dynamics of appearance-based facial expression representations may be utilized to distinguish between distinct emotions, according to experiments using two difficult publicly accessible datasets.

To extract facial image characteristics and reliably identify the pain, an ensemble deep learning framework (i.e., Ensemble Deep Learning Model, EDLM) is suggested in [41]. It includes fine-tuned, three-stream hybrid deep neural networks. To extract features from photos, the VGG-Face is optimized, integrated, and used. The outputs of the three-layer hybrid CNN and recurrent neural network method are then combined to create image-classified features that may be used to categorize different pain intensities. Patania et al. [42] presents a cutting-edge method for identifying pain by analyzing facial expressions. A Graph Neural Network (GNN) is used to inherit a collection of fiducial points that are automatically monitored on subject faces. It is achieved utilizing the publicly accessible BioVid dataset. Past studies have employed deep learning and machine learning to either identify pain or quantify its intensity. In [43], a refined method for detecting pain from facial expressions using pre-trained data-efficient image converters and distillation (Deit) is suggested. UNBC McMaster Shoulder Pain and BioVid Heat Pain are two publicly accessible databases that are used to assess the effectiveness of the suggested design. Comparing the suggested method to the state of the art, the first findings are encouraging.

Witherow et al. [44] proposes a novel deep domain adaptation approach called FACE-BE-SELF (FACial Expressions fusing BEtaMix SElected Landmark Features) to classify facial expressions by aligning their distributions in a shared latent space. On the other hand, using a distinct mixture of beta distributions, FACs are effectively selected based on expression, domain, and identity correlations. The FACE-BE-SELF approach is unique in its capability to simultaneously adapt to adult and child domains, resulting in a unified expression representation.

De Melo et al. [45] introduced the Decomposed Multiscale Spatiotemporal Network (DMSN) to provide an efficient and accurate solution for automatic depression detection and pain estimation through video-based facial analysis. To overcome the high computational complexity and parameter count of traditional 3D-CNNs, the authors developed a method to decompose multiscale spatiotemporal feature extraction into three variants that capture the gradual variations seen in depression and rapid facial changes associated with pain. Table 1 summarizes facial expression-based approaches for pain monitoring.

**Table 1:** Pain monitoring using facial expressions.

Paper	Dataset	Feature	Model	Stimuli	Subjects	Performance Metrics	Result
Kaltwang S’12 [28]	UNBC-McMaster Shoulder Pain Database	PTS, DCT, LBP	RVR	Arm movement, Shoulder rotations	25	MSE, CORR	1.804, 0.502
Hammal Z’12 [29]	UNBC	CAPP	SVMs	Arm movement, Shoulder rotations	25	F1, CR, PR	60, 80, 70
Dey Roy’16 [27]	UNBC	Gabor filtering	SVMs	Arm movement, Shoulder rotations	25	Accuracy	82.43%
Rodriguez P’17 [32]	UNBC	VGG	LSTM	Arm movement, Shoulder rotations	-	MSE, AUC, MAE, Accuracy	0.74, 93.3, 0.5, 97.2%
Chen’17 [30]	UNBC	HOG, HOG-TOP	SVM	Arm movement, Shoulder rotations	–	Accuracy, F1-score	0.86, 0.542
Lo Presti’17 [40]	UNBC	Haar and Gabor	AdaBoost	Arm movement, Shoulder rotations	–	Accuracy	0.59
Tavakolian’18 [33]	UNBC	CNN	Deep binary encoding network	Arm movement, Shoulder rotations	-	MSE, PCC	0.69, 0.81
Haque’18 [39]	MIntPAIN/ UNBC	VGG	LSTM	Electrical/shoulder pain	20	Accuracy	36.55
Bargshady’19 [34]	UNBC	VGG	RNN	Arm movement, Shoulder rotations	–	Accuracy	92.5%
Tavakolian’19 [37]	UNBC	–	CNN	Arm movement, Shoulder rotations	–	MSE, PCC	0.32, 0.92
Bargshady’20 [35]	UNBC	VGG	EJH-CNN-BiLSTM	Arm movement, Shoulder rotations	–	AUC, Accuracy, MSE, MAE	88.7%, 85%, 20.7, 17.6
Tavakolian’20 [38]	UNBC/BioVid	Unsupervised learning	Siamese Network	Shoulder/heat pain	–	MSE, PCC	0.92, 0.78
Bargshady’20 [41]	MIntPAIN/ UNBC	VGG	EDLM	Electrical/shoulder pain	–	AUC, Accuracy, MAE, MSE, F-score	90.5, 86%, 0.103, 0.081, 86.2%
El Morabit’21 [31]	UNBC	3D, 2D and 1D CNN	Multiple state-of-the-art methods	Arm movement, Shoulder rotations	25	MSE	–
Huang’21 [36]	UNBC	Spatiotemporal, geometric	Hybrid Network	Arm movement, Shoulder rotations	–	MAE, MSE, PCC	0.40, 0.76, 0.82
Pataiu’22 [42]	BioVid	Approximate, Sample, Permutation and SVD Entropy	Graph neural network	Heat pain	1245	Classification Accuracy	0.73
S. E. Morabit’22 [43]	UNBC/BioVid	–	DeiT	Shoulder/heat pain	–	Accuracy	84.15%/ 72.11%
Witherow’24 [44]	CK+/Aff-Wild2/ CAFE/ ChildEFES	geometric and texture-based	FACE-BE-SELF	Posed, Spontaneous	123/154	F1-score	84%
De’24 [45]	AVEC2014/ UNBC/BioVid	CNN	DMSN	Heat pain/Shoulder pain and depression	25	MSE, MAE, PCC	0.38, 0.35, 0.83

Model (Convolutional Neural Network (CNN), Support Vector Machines (SVMs), Random Forest Regression (RFR), Relevance Vector Regression (RVR), Support Vector Regression (SVR), Data-Efficient Image Transformers and Distillation (Deit) and Ensemble Deep Learning Model (EDLM), Long Short-Term Memory Networks (LSTMs)). Performance metrics (Mean Absolute Error (MAE), Mean Squared Error (MSE), Pearson Correlation Coefficient (CORR), Probability of Correct Classification (PCC), Features (Histogram of Oriented Gradients (HOG), Facial Landmark Points (PTS), Discrete Cosine Transform (DCT), Local Binary Pattern (LBP), Canonical Normalized Appearance of the Face (CAPP), Visual Geometry Group (VGG)).

#### 4.1.2. Audio/Speech Prosody-Based Approaches

While early research in audio-based pain assessment primarily focused on newborns due to their distinct vocalizations, the majority of literary works in this field have concentrated on infants since older patients sometimes stifle their cries or weeps because of pain. In [46], a method for automatically identifying newborn cries is presented. Cries are examined using spectrograms. Then, using CNN, it is divided into three categories: drowsy, in pain, and hungry. In another attempt to classify pain screams, the country Hungary used an algorithm based on an improved fuzzy model [47]. Convolutional Restricted Boltzmann Machine (ConvRBM)-based unsupervised auditory filter bank learning is suggested in [48]. Only a few articles have used audio to evaluate pain, except the newborn scream domain.

A pain identification technique is suggested to distinguish between speech data with and without pain. Ref. [49] showed that there is a significant correlation between self-reported pain level and speech biosignal characteristics. The accuracy of pain diagnosis may be increased by automated pain assessment based on paralinguistic speech cues. The Dusseldorf Acute Pain Corpus, a unique audiovisual pain database, is shown in [50], where patients underwent a cold presser pain induction. Support vector machines and long short-term memory recurrent neural networks are used to categorize pain into three categories (LSTM-RNN). A significant challenge in analyzing audio data for pain detection is distinguishing the sounds of interest from background noises, which can originate from medical equipment, other individuals, or incidental occurrences within the environment.

Andrew [51] proposed a real-time framework that helps with intelligent care and independent living using voice-based pain level classification. It uses convolutional neural networks that extract various features from non-verbal and verbal vocalizations of the patients to measure pain level, which could be low, moderate, or high. This framework is suitable for home-based monitoring in low-resource environments. Alhudhaif [52] proposes a lightweight recurrent model based on deep learning that can operate in real-time and can classify pain level using raw audio features. In this model, raw audio features are converted into a spectrogram, and a three-layer bidirectional Gated Recurrent Unit (GRU) is applied. This end-to-end architecture outperformed classical machine learning baselines. Table 2 summarizes audio-based pain monitoring approaches.

#### 4.1.3. Iris Tracking-Based Approaches

Due to a paucity of publicly accessible datasets, there are few attempts at employing iris tracking to measure pain in the literature. Fear of pain is a key sign that there is pain present. Using eye-tracking techniques, the consequences of fear of pain (FOP) are investigated [53]. While their eye movements were being recorded, 177 undergraduate students were given comforting information regarding the cold presser task. According to the findings, being initially alert to affective pain was a strong predictor of reporting pain on the cold presser more rapidly. However, an over-reliance on self-reported pain as the sole ground truth in such studies can introduce subjective bias, potentially limiting the generalization and objectivity of the findings.

Technical challenges in iris tracking include the variability of lighting conditions, which can affect image quality and detection accuracy, and the impact of rapid eye movements or blinks that can lead to data loss or misinterpretation. The suggested iris tracking technique makes use of the Isophote Curve and the Daugman Algorithm’s advantages to find the iris. Since the primary step involves correctly locating the iris, this method can be utilized for pain monitoring. Through the monitoring of pain intensity, the proposed system can be very helpful in spotting early signs of medical issues [54]. Eye tracking could be used to measure the degree of ocular motility disruption brought on by structural brain injury and concussion.

To assess the level of discomfort in brain-damaged people, an eye movement tracking method is given in [55]. All 75 trauma patients underwent linear regression analysis, which produced good findings for tracking brain damage pain. A machine learning-based approach that uses saccadic eye movements to characterize concussion phases has been created [56]. An analysis is done on a dataset of 34 mild traumatic brain injuries. Implemented and trained in chosen characteristics for classification was an ensemble model made up of several random forest classifiers. To discern nonlinear patterns in saccadic eye movement, more regression is employed to examine the data. During the cold presser test, a second automated evaluation of pain severity is carried out using a customized Bidirectional Long Short-Term Memory Recurrent Neural Network (BiLSTM RNN), an ensemble of BiLSTM, RNN, and Extreme Gradient Boosting Decision Trees (XGB) [57]. The original signals are enhanced with the tonic and phasic components of the deconstructed EDA signals. The analysis also includes a concatenation of fourteen knowledge-based features taken from EDA signals and the deep learning feature representations. This study demonstrated how deep learning may help us estimate pain by generating deep representations of physiological signals that go beyond domain experts.

A framework for the estimation of pain by Othmani et al. [58] has been put forth that utilizes eye tracking along with high-end machine learning techniques. DeepLabV3+ is used for high-precision eye area segmentation with an IoU score of over 99%. Physiological parameters including specific physiological parameters like pupil size, blinking rate, and saccade velocity are captured through VGG16. SHAP analysis highlighted pupil size and blink rate as the most critical predictors of pain level. A study was done by Zhang et al. [59] in an attempt to create machine learning models for the intelligent assessment of scientific creativity, aiming to replace labor-intensive and subjective manual scoring processes. The study involved students who participated in a scientific relatedness judgment task to map knowledge structures and a scientific creativity test.

The researchers extracted five key input features, including four semantic network parameters. The Clustering Coefficient and blink duration were identified as the most significant predictors. Table 3 presents a summary of iris-based pain monitoring approaches.

### 4.2. Multimodal Approaches

Audio is frequently used in combination with other modalities in the literature. Thiam et al. [63] used audio signals containing the activity of multiple modalities, e.g., video, electrodermal, electrocardiography, electromyography, and respiration. The data of these modalities is analyzed on a broad spectrum followed by fusion architectures that combine extracted descriptors. An automated pain assessment system is presented [64] to analyze infants crying and pain indicators. The results indicate that performance is significantly higher when audio is combined with other modalities, e.g., body motion, vital signs, and facial expressions. Another author used audio and video recordings of patients to observe expressive behaviors of pain among patients [61]. It used audio, facial expressions, clinically related data, and physiological vital signs (heart rate, blood pressure) in the pain assessment system. A machine learning-based model classifies pain in three different intensity levels after detecting their presence. To observe pain perception and variance among various patients with chronic low back pain, a novel residence pain measurement system is described. Heart rate signals, speech traits, and facial expressions are used to study the autonomous nervous system and audio–visual features [65].

Jiang [66] suggested a neural network approach that incorporates pain sensitivity into a phase of personalized feature fusion and dynamic feature attention. The results indicate that dynamic attention enhanced prediction recall using soft selection of physiological features and improved the precision using pain sensitivity fusion. The proposed approach is verified using BioVid Heat Pain data, and it has been noticed that the adaptation capability of the approach works better for a different pain protocol using the high accuracy of time continuity in pain detection. A hybrid multimodal AI model has been created for the purpose of enhancing the intensity and accuracy of pain detection using the integration of facial gestures and paralanguage [67]. Utilizing convolutional neural networks (CNNs) for the detection of spatial facial gestures and recurrent neural networks with long short-term memory (LSTM) for vocal features, the strategy overcomes some of the problems particularly with regards to non-communicative populations. With its groundbreaking strategy, it is essential to ensure Explainable AI (XAI) integration, particularly for the benefit and knowledge of medical professionals. A machine learning-based system is proposed for the objective measurement of the intensity of acute pain using various physiological responses, i.e., ECG, GSR, and EMG [68]. It tries to eliminate the problems seen in the subjective measurement methods used in the past, making them unfeasible for patients not able to communicate, like infants or patients with dementia. Various classifiers have been trained using the BioVid database. The results prove that this system provides a cost-effective solution for the measurement of acute pain intensity without the need for costly neuroimaging techniques. Table 4 provides a summary of multiple modality-based pain monitoring approaches.

### 4.3. IoT-Based Approaches

#### 4.3.1. Wearable Device/Body Sensor-Based Approaches

Many researchers have started to explore the possibility of using technology as unbiased instruments to contextualize pain as well as physical functions in settings where people can live freely. However, it is crucial to acknowledge the potential for bias in sensor data acquisition and algorithmic interpretations, necessitating rigorous validation to ensure their objectivity and reliability. The majority of the research that has been carried out is only concerned with functional outcomes and physical activity as determined by a wearable accelerometer. Several research have shown the presence of a positive relationship between pain scores and indicators obtained by means of wearable technologies, physical activity, and physical function. Additionally, research employing wearable technology to monitor physiological signals and link them to pain in free-living contexts is scarce [70]. Furthermore, the collection and use of sensitive health data through wearable devices raise significant privacy and security risks that must be carefully addressed through robust ethical frameworks and data protection protocols.

However, there is a documented correlation between physiological signals that may be detected by wearable devices and pain. The type of body signals which can be monitored include heart, brain, muscle, respiratory, blood volume, skin temperature, and accelerometer data [71].

To overcome the issues related to verbal methods, a biopotential and behavioral parameter-based automated pain recognition system is presented [72]. Individuals are exposed to uncomfortable heat stimuli for this objective under supervised circumstances. The mathematical groups of amplitude, frequency, stationarity, entropy, linearity, and variability allowed for the extraction of 135 characteristics. Electromyography corrugator peak to peak, corrugator Shannon entropy, and heart rate variability are the most discriminating characteristics. In the article, multimodal detection is used (i.e., simultaneous data collection on electromyogram [EMG] including zygomaticus, corrugator, and trapezius, skin conductance level [SCL], and electrocardiogram [ECG]). The automated feature patterns with the best rate of identification for quantifying pain statistically and individually were sed. Another study looked at phasic pain induction and pain detection using EEG [73]. The coldness was used to induce phasic pain, after which dynamic EEG data were examined using the Recurrence Quantification Analysis (RQA) method. Finally, the neural network classifier was used to achieve an accuracy of 95.254% in identifying and classifying pain and non-pain states. The simulation’s outcomes proved that it is possible to observe brain activities under pain. An algorithm is created to recognize pain in real-time and in outdoor settings [74]. Using the cold presser test, 41 human individuals were subjected to severe discomfort while having their electrocardiograms recorded. To construct logistic regression classifiers, various characteristics of time, frequency, and nonlinear domains of respiratory and heart rate variability data were calculated. The derived pain algorithms might be used to measure intense pain utilizing information from a variety of sites, including pulse plethysmography information from an increasing number of consumer wearables or ECG data in clinical settings. Functional near-infrared spectroscopy (fNIRS) can be used for pain detection, according to the latest neuroimaging research. This method of brain imaging enables continuous, non-invasive assessments of variations in cortical hemoglobin concentration. A method for detecting the existence of pain using Bayesian hierarchical modeling with wavelet characteristics was developed in [73] by focusing on fNIRS signals only obtained from the prefrontal brain, which may be accessed without being noticed. By considering the variation in pain responses between participants, this method enables the personalization of the inference process. This paper shows the need for a tailored strategy and supports the use of fNIRS for pain evaluation.

Gungormus et al. [75] developed a semi-automatic mHealth system that integrates wearable devices and psychological assessments to predict pain thresholds in elderly individuals with preclinical chronic pain. The physiological measures are combined with psychological data on perceived stress and stress vulnerability. The pain thresholds are predicted using multiple linear regression.

Ayena et al. [76] carried out a review article which emphasized the possibilities for the prediction of chronic pain by monitoring real-time physiological factors such as heart rate, heart rate variability, and physical activity levels. The review found that predictive modeling remains significantly underutilized, with most current research focusing on data correlation rather than the proactive anticipation of pain episodes. Among the models used in the analysis presented in the paper, Random Forest and multilevel models showed good consistency in their results, while complex models such as CNN-LSTM have been experiencing difficulties in terms of both computational costs and data quality. Table 5 presents a summary of this section.

#### 4.3.2. Wireless/Remote Pain Monitoring

A thorough review of the research from 2000 to 2018 that describes the application of one or several IoT-enabling techniques for diagnosis is done in [81]. Under the IoT philosophy, IoT-enabling technologies have typically been utilized on their own rather than in combination. Security and privacy concerns, as well as the challenges of pain assessment itself, have prevented the widespread adoption of IoT-based strategies for diagnosis and/or management. For this sector to advance, engineers and healthcare professionals must work well together. Table 6 demonstrates that smartphones [82,83,84,85,86,87,88,89] and other smart mobile devices are the most popular tool for IoT-based pain monitoring. Particularly, smartphone apps, also known as apps, are becoming increasingly well-liked among people of all ages worldwide. In [90,91,92], just a few cloud server-based remote monitoring and processing systems are covered. The possibilities of IoT connection protocols are assessed to deliver the physiological data stream to a cloud server and FOG computing [93,94]. Table 6 presents a summary of this section.

## 5. Public Datasets for Adults

There are several datasets available for automatic pain monitoring. In this section, we categorized these datasets by the subject type, pain type, pain intensity level, and modality level of patient. Here are the details of the datasets being used in the automatic pain monitoring techniques.

### 5.1. UNBC-McMaster Shoulder Pain [95]

**Figure 3:**
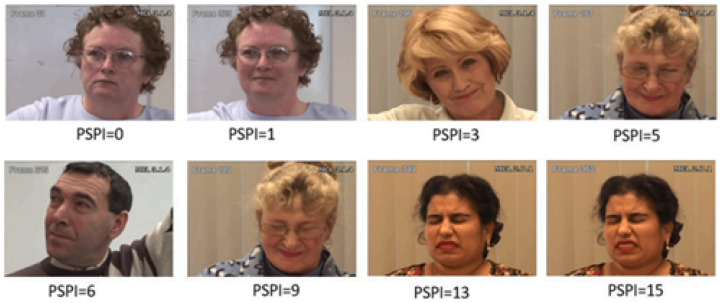
Image frame sample of UNBC-McMaster Shoulder Pain Archive database [35].

This dataset has records of twenty-five adults with shoulder pain. It is divided into four sections overall. The first part contains videos of spontaneous face expressions. The second part contains coded frames with a Facial Action Coding System (FACS). The third part has frame-by-frame scores for associated pain, observer measures, and self-report at the sequence level. The last part contains landmarks of a 66-point Active Appearance Model (AAM). Prkachin and Solomon Pain Intensity (PSPI) score is used to code each frame of the database at a 0–15 scale. In this database, the number of images with no pain label is higher than number of images with pain labels. The images with PSPI > 6 are very few. Therefore, the trained model can be biased toward no pain. Researchers should consider employing data augmentation techniques or weighted loss functions during model training to mitigate this inherent class imbalance. This face should not be ignored while using this database. Figure 3 shows samples of the UNMC-McMaster Shoulder Pain database with associated PSPI scale.

### 5.2. BioVid [46]

In the BioVid database, data of 90 healthy subjects is included from three different age groups. The first group has subjects in the 18–35 years group, the second is 36–50 years, and the last group has 51–65 years. All three groups have equal numbers of men and women. This database is multimodal. Data of 87 subjects is publicly available. The reason for the exclusion of data from the remaining three subjects is not specified in the publicly available documentation. Heat pain is induced at four intensity levels for experiments. In total, 17,300 5 s videos are recorded with 25 fps. For each level, twenty experiments are performed. It contains biomedical signals (Galvanic Skin Response (GSR), skin conductance level (SCL), electromyography (EMG), and electrocardiography (ECG)) and frontal videos.

### 5.3. BP4D-Spontaneous [96]

The BP4D-Spontaneous dataset fulfills the need of three dimensional spontaneous facial videos to analyze behavior during pain monitoring. Although two dimensional facial videos are available, their analysis is complex due to differences in multiple dimensions. This dataset has spontaneous videos of facial expressions and emotion elicitation videos (3D, colored). It contains records of 41 healthy adults in the age range of (18–29) years. The pain is induced using a cold pressor task. The cold pressor task involves immersing a limb in cold water to elicit a controlled pain stimulus, allowing for standardized pain induction across subjects.

### 5.4. BP4D+ [97]

In the BP4D+ database, 140 healthy adults participated in performing a cold presser task. The subjects are 18–66 years. It is a multimodal dataset. Different modalities including video of face (thermal, 3D, color), heart rate, respiration rate, blood pressure, and electrocardiogram are recorded in this database. Similar to BP4D-Spontaneous, the cold pressor task was utilized to induce pain, ensuring consistent and reproducible pain stimulus for data collection.

### 5.5. EmoPainA [98]

The EmoPain dataset includes data of 50 subjects in total. Twenty-two subjects are chronic lower back pain patients with an average age of fifty years. Twenty-eight subjects are healthy adults with average age = 37 years. The healthy subjects performed different physical exercises during the experiments. It is a multimodal dataset. The video, audio, motion capture, and surface EMG (sEMG) of each patient and health controls are included in the dataset.

### 5.6. MIntPAIN [40]

**Figure 4:**
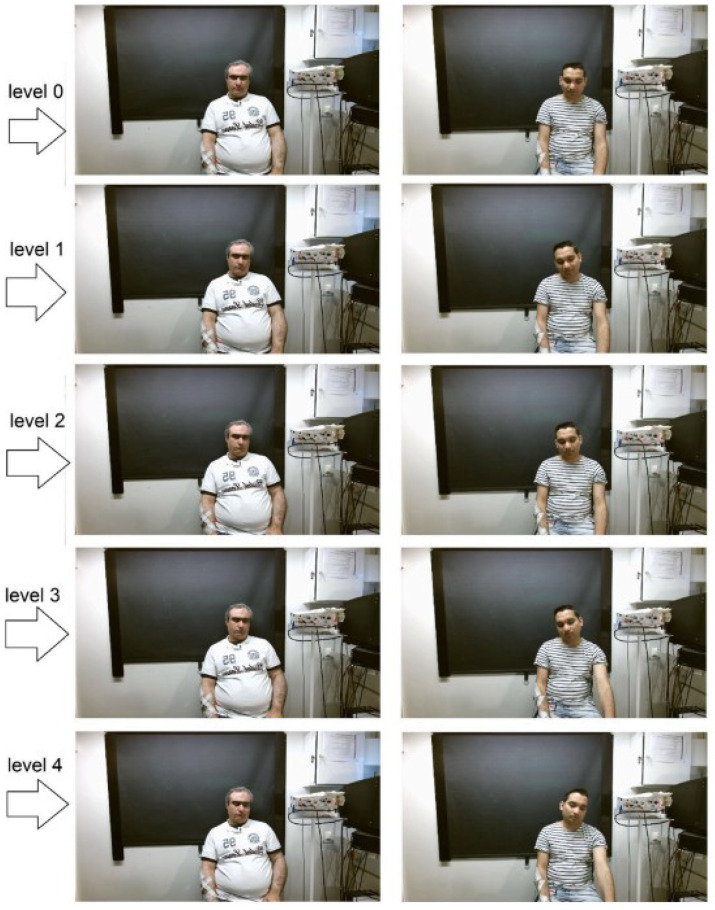
Samples of selected dataset of MIntPAIN database [40].

The MIntPAIN database includes 20 healthy subjects. The pain is induced with electrical stimulation. There are four intensity levels (0–4) of pain where level 0 is no pain level and level 4 is the highest intensity pain level. Each subject belongs to weight (50–110) kg, height (1.6–2) meters, and age (22–42) years. Each subject performed two trails; one with no pain and another with one reading for all four intensity levels. During EMG, some sweeps are missing due to patients talking to other patients. There is a total of 9366 colored videos. There is an average of 20.07 frames in each sequence for RGB. The duration of each sequence is 1–10 s. Figure 4 shows examples of cropped database face samples.

### 5.7. SenseEmotion Database [69]

The SenseEmotion database is created for pain and emotion-recognition. In this database almost 45 healthy people’s data is collected with an average age of 26. This data includes 30 min of multimodal sensory data. The three modalities used in this data collection are biopotentials, facial region camera images, and audio signals. Heat and sound stimulation are used to elicit pain. It includes videos of face, audio, EDA, ECG, sEMG, and RSP.

### 5.8. X-ITE Pain [99]

The X-ITE pain dataset consists of records of 134 healthy adults, 18–50 years, when they had 24k phasic pain and 804 tonic pains. Heat stimulation is used for phasic pain and electrical stimulation is used for tonic pain each with three intensities. Videos of face (color, thermal) and body and audio, ECG, SCL, EMG, and sEMG are recorded for all subjects.

Table 7 shows a comparative study of the dataset using certain parameters like the subject type, pain type, pain intensity level, and modality level of the patient.

## 6. Limitations, Challenges and Future Directions

The idea of automatic pain recognition and assessment has been an interesting research topic over the last decade, gathering research activities related to the development of data acquisition and computation. Here, data acquisition comprises a set of issues that can be related to hardware devices and sensors, as well as experiments and information processing. The computation deals with the processing of gathered data with a focus on detecting pain and its intensity level. It deals with issues such as, e.g., data filtering, model building, classification, and validation of results.

There are certain inherited challenges in automatic pain monitoring using the various technologies. The first major challenge is pain subjectivity, as pain is a very subjective experience and it is very difficult to quantify in most cases. The next challenge is the variability in the intensity and duration of the pain; it is very hard to make a generalized model to measure pain intensity. Another major challenge is pain heterogeneity, as pain can be classified into different forms such as acute, chronic, neuropathic, or psychogenic, which makes automatic recognition more challenging. Pain modalities are another challenging factor for the automatic monitoring of pain. As data plays an important role in model development for automatic monitoring of pain, the lack of pain-related data availability is another significant challenge in making pain monitoring systems. Along with all these challenges, ethical consideration is another significant challenge as it violates the data privacy and confidentiality of patients. For instance, the continuous collection of physiological data from individuals raises concerns about who has access to this sensitive information and how it is protected from unauthorized use or breaches. Mitigation strategies involve implementing robust anonymization techniques, securing data storage with advanced encryption, obtaining explicit and informed consent from participants, and adhering to strict regulatory guidelines like GDPR or HIPAA to ensure patient autonomy and data integrity.

In addition to the challenges discussed above, several key factors must be considered for the effective development of automatic pain monitoring systems. The most important factor is high-quality data. The availability of large, labeled datasets helps in developing these systems, using advanced technologies like sensors and wearable devices, and capturing facial, vocal and physiological responses help to build these systems. The selection of effective machine learning and deep learning algorithms plays a crucial role in building effective systems. The development of standardized pain assessment protocols can help in building the pain monitoring system. Ethical considerations such as privacy and confidentiality of data ensure patient and health practitioner confidence in using these systems. Computer vision-based approaches can be used to recognize different types of pain based on facial expressions. Using computer vision techniques, such as facial landmark detection and facial expression recognition, it is possible to automatically detect and classify pain based on these scoring systems. For example, by analyzing changes in facial muscle movements, such as furrowed brows or tightly clenched jaws, a computer vision system can infer the presence and severity of pain. Computer vision-based approaches are not always accurate in recognizing pain, particularly in individuals with certain medical conditions or cultural differences in facial expressions. Therefore, they should be used as a complementary tool to other methods of pain assessment, such as self-reporting and physiological measures.

Audio-based pain monitoring techniques involve the analysis of sounds such as moaning, crying, speech prosody, and other non-verbal sounds. These approaches have several limitations. These are extremely vulnerable to background noise and interference, which is frequently encountered in clinical settings. Audio-based pain monitoring techniques also require patients to vocalize their pain. Therefore, they cannot be employed for patients who do not speak due to medical issues or even for stress or anxiety patients, as there is a possibility of confusion between expressions of pain and emotional stress.

The effectiveness of iris-based pain monitoring techniques relies on the features that are monitored, such as the level of pupil dilation, texture of the iris, and patterns of eye movements. However, the effectiveness of this technique is affected by a few challenges. Iris-based monitoring techniques are very sensitive to changes in illumination, reflections, and the position or orientation of the camera. Iris features can be obscured by eye lids, eyelashes, contact lenses, or partially closed eyes. Furthermore, differences in eye features among individuals due to age differences, variations in eye colors, and health differences also contribute limitations to this system.

IoT (Internet of Things)-based approaches can be used to recognize different types of pain based on physiological measurements and other sensor data. For example, wearable devices such as smartwatches, fitness trackers, and medical sensors can collect data on heart rate, blood pressure, skin temperature, and other physiological parameters that can be used to detect pain. In addition to physiological data, IoT devices can also collect other types of data that may be associated with pain, such as movement patterns, sleep quality, and medication usage. By analyzing these data streams using machine learning algorithms, it is possible to automatically detect and classify different types of pain. However, it is important to note that IoT-based approaches to pain detection are still in the early stages of development and may not be as accurate as other methods of pain assessment, such as self-reporting. Additionally, there are ethical and privacy concerns related to the collection and use of sensitive health data, which must be carefully considered when designing and deploying IoT-based pain detection systems.

Multimodal approaches integrate information from different modalities, so it is possible to capture different aspects of pain and reduce the impact of individual differences or measurement error in any one modality. Multimodal pain monitoring has the potential to provide more accurate and reliable pain detection, especially in cases where self-reporting is not possible or reliable. However, it also requires careful integration of data from different sources and the use of sophisticated machine learning algorithms that can handle complex and heterogeneous data streams.

**Figure 5:**
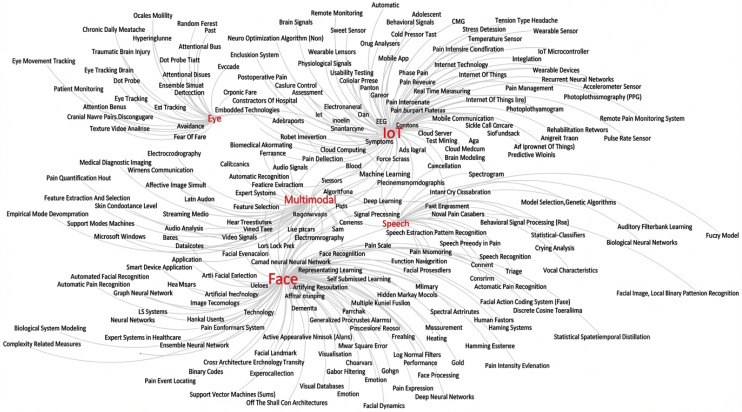
Keyword map for taxonomy of automatic pain monitoring techniques.

Figure 5 shows a keyword map for the taxonomy of automatic pain monitoring techniques. This map was generated by analyzing the keywords extracted from the abstracts of articles included in Section 3 of this survey. A total of 66 shortlisted abstracts were analyzed, and keywords were extracted from these studies to construct a keyword co-occurrence network. Figure 5 is generated using the Gephi software to visualize the relationships among keywords related to automatic pain monitoring techniques. Each keyword was treated as a node, and connections between keywords were established based on their occurrence within the same paper. The network was visualized using the ForceAtlas2 layout algorithm, which helps to spatially organize related keywords into clusters. It shows five clusters (red color) belonging to three main categories: computer vision, multimodal, and IoT. More precisely face, speech, and iris-based approaches lie under the computer vision category. The edges emerging from each cluster show a keyword at the end (black color). The size of the cluster is a measure to the number of research articles which refer to it as a keyword. Here, face is the most frequently used keyword in selected research articles and other keywords used in this domain include representation learning, convolutional neural network, videos, pain event detection, self-supervised learning, 1D/2D/3D convolution, etc. To interpret the map, larger clusters and thicker edges indicate a higher prevalence and stronger interconnections of those keywords and research areas, guiding researchers to dominant themes and potential interdisciplinary opportunities.

The keyword map helps researchers refine their studies by identifying frequently used keywords within each domain. This map shows that during the last decade, IoT and facial expression, multimodal, and speech- and eye-based automatic pain monitoring approaches are explored frequently in descending order. This is why eye clusters are the smallest, and the face cluster is the largest. The reason why eye-based approaches got less attention lies in the high error rate in tracking the iris area and analyzing it for pain intensity. It is also computationally expensive as first the location of the eye needs to be found on the face and then the iris is tracked, so researchers prefer face expression-based approaches, which have more room to decode the expressions for pain intensity.

The advancement of automated pain monitoring will be determined by overcoming the division between algorithmic precision and clinical utility. Future directions must emphasize the construction of effective multimodal approaches that combine facial data with neurophysiological and bio-behavior to account for the complex variability associated with pain. For integration into clinical practice, these systems must move beyond a “black box” model by incorporating Explainable AI (XAI) methodologies that provide transparent assessments. Ethical issues related to patient data privacy and informed consent with non-communicative patients need effective frameworks to support a transition toward precise pain management.

## 7. Conclusions

Pain is a complex, subjective experience that originates in the brain that profoundly influences an individual’s well-being and drives behaviors aimed at relief. It is very important to understand this phenomenon clearly, especially in patients who have speech or language impairment and infants. This survey systematically reviewed recent methods for pain monitoring. It also includes publicly available datasets that can be used in the development of pain monitoring systems. This paper presents a taxonomy of pain monitoring methods that not only organizes existing approaches but also clarifies their relationships, thus supporting researchers in identifying gaps and emerging directions in the field.

The paper provides a comparative analysis of the strengths and limitations of key pain assessment modalities, including physiological signals, facial expressions, vocalizations, and behavioral patterns. Overall, this survey analyzes the current state of automatic pain monitoring methods, offering key insights into modality selection, system design, and deployment challenges.

Future research should explicitly address methodological limitations and ethical concerns, particularly data privacy and model generalizability. Addressing these issues is essential for building trust and ensuring the responsible deployment of pain monitoring systems in real-world clinical settings. There is a need to develop robust and explainable AI models that integrate multimodal data, validated with diverse patient populations and governed by ethical frameworks for data collection. These directions are critical for translating automatic pain monitoring methods from research prototypes into trustworthy clinical systems.

## Figures and Tables

**Table 2:** Pain monitoring using audio-based approaches.

Paper	Dataset	Feature	Model	Stimuli	Subjects Type	Performance Metrics	Result
A. Rosales-Perez’ 15 [47]	Baby Chillanto	304, MFCC	GSFM	-	542/Infants	Accuracy, AUC	97.96, 98.89
Chuan-Yu’ 16 [46]	National Taiwan University	-	CNN	-	2000/Infants	Prediction probability	-
Oshrat’16 [49]	Interview-based dataset	1582, openSMILE	SMO	spinal cord and/or brain injuries, pain	27/Adults	CCI	0.7725
Sailor ’18 [48]	Baby Chillanto	39, ConvRBM	GMM	-	192/Infants	MCC, F-measure, J-statistics	0.993, 0.995, 0.99
Zhao’19 [50]	Duesseldorf Acute Pain Corpus Audiovisual database	MFCC, deep spectrum	SVM, LSTM-RNN	cold pressor pain induction	80/Adults	UAR	42.7%
Alhudhaif’25 [52]	TAME	Log-Mel spectrogram features	GRU	cold pressor pain induction	51	Accuracy	75.36–86.8%
Andrew’26 [51]	TAME, VIVAE	MFCCs, pitch, formants, spectral energy and centroid, zero-crossing rate	CNN	physical discomfort	-	Accuracy, latency	72.74%

Model (Support Vector Machine (SVMs), Recurrent Neural Network (RNN), Long Short-Term Memory Recurrent Neural Networks (LSTMs), Convolutional Neural Network (CNN), Gaussian Mixture Model (GMM), Genetic Selection of a Fuzzy Model (GSFM), Sequential Minimal Optimization (SMO), Unweighted Average Recall (UAR)). **Performance metrics** (Matthews Correlation Coefficient (MCC), Correctly Classified Instances (CCIs)). Features (Computational Paralinguistic Challenge (ComParE), Mel-Frequency Cepstrum Coefficients (MFCCs)).

**Table 3:** Pain monitoring using iris tracking.

Paper	Dataset	Feature	Model	Stimuli	# Subjects Type	Performance Metrics	Result
Yang’13 [60]	self	-	ANOVA	cold pressor pain induction	756/Adults	Orienting and Maintenance Bias Scores	-
Christina’14 [61]	self	-	ANOVA	Chronic Pain	46/Adults	Proportion of initial fixation location, duration, visits and fixation duration	71.0
Samadani’15 [55]	Self	-	LR	-	64/Adults	AUC, Sensitivity, Specificity, Positive/Negative predictive	0.761, 0.62, 0.78, 0.36, 0.91
Michael’16 [54]	Self	Texture-based	Modified Daugman, Isophote Curved Algorithm	-	8/Adult	Accuracy	88.80%
Sharpe’16 [53]	Self	-	Regression	Cold presser	107/Adults	Tolerance	-
Fashler’16 [62]	self	-	-	Chronic pain during exposure to injury-related pictures	113/Adults	Frequency of gaze (N), Attentional maintenance and phases	129.53, 0.185, 0.136
Tirdad’21 [56]	self	Statistical features	RF	-	34/Adults	Accuracy	-
Fatemah’22 [57]	self	EDA	RNN	Cold pressor	29/Adults	Precision, Recall, F1-Score	0.87, 0.84, 0.86
Othmani’25 [58]	self, UNBC	Pupil size, blink rate, saccade velocity	XGBoost, RF, SVM	visual indicators of pain	-	Accuracy	99.5%
Zhang’25 [59]	Self	KNN, RF, GBDT, ANN	-	-	68	Accuracy	90–100%

Model (Random forest (RF), Recurrent Neural Networks (RNNs), Linear Regression (LR)). Feature abbreviations include Electrodermal Activity (EDA), K-Nearest Neighbors (KNNs), Random Forest (RF), Gradient Boosting Decision Tree (GBDT), and Artificial Neural Network (ANN).

**Table 4:** Pain monitoring using multiple modalities.

Paper	Dataset	Feature	Model	Stimuli	# Subjects Type	Modalities
Tsai’16 [61]	Chang Gung Memorial Hospital	Low-level descriptor	SVM and RF	chest, abdominal, lower-back, limbic pain, and headaches	182/Adults	Audio, facial, vital signs
Thiam’ 17 [63]	SenseEmotion Database [69]	Geometric, Head Pose, Appearance-based	RF, LDA	heat stimulation	45/Adults	Audio, facial and Vital Signs
Zamzmi’17 [64]	Self	Strain, motion, descriptive Statistics	SVM	heel lancing and immunization	18/Infants	Audio, facial, body motion, State of arousal and Vital Signs
Keskinarkaus’22 [65]	Northern Ostrobothnia Hospital District, Oulu, Finland	LBP, facial landmarks, Spatio-temporal	SVM	Low back pain	14/Adults	Facial, Audio analysis, Heart
Jiang’23 [66]	SpaExp, BioVid	GSR, ECG, HRV	NN, RNN	electrical, heat pain	117/Adults	GSR, ECG
Gutierrez’24 [67]	Own dataset, BioVid	Facial, Auditory	LSTM, CNN	social interaction, heat pain	200/Adults	Facial, paralanguage
Albahdal’24 [68]	BioVid	Physiological features	Random Forest, SVM, LR, DT, NB, KNN	induced heat pain	86/Adults	ECG, GSR/EDA, EMG

**Features** (Local Binary Pattern (LBP)). Model (Random Forest (RF), Linear Discriminant Analysis (LDA), Support Vector Machine (SVM), Galvanic Skin Response (GSR), Electrocardiography (ECG), Heart Rate Variability (HRV)).

**Table 5:** Pain monitoring using wearable device/body sensor-based approaches on self-datasets.

Paper	Sensors	Feature	Model	Stimuli	Subjects Type	Performance Metrics	Result
Walter’14 [72]	Skin conductance level/EEG/ECG	135, electromyography, entropy, heart rate variability	SVM	heat stimuli	90/Adult	Accuracy	-
Seok’19 [77]	PPG	8, basic, normalized and dynamic	multiple LR	-	78/Adults	Accuracy, AUC	0.752, 0.825
D. Lopez’19 [73]	Heartbeat, respiration and blood pressure	Scalogra, frequency oscillation	Wavelet Transform, Bayesian Modeling	Resting-state session, brush session, electrical stimulation	43/Adults	Accuracy, F1	0.81, 0.73
Wang’20 [78]	EEG	-	k-NN, SVM, LDA, LR	-	29/Adults	PCA, MDS, AE	50.9, 56.8, 71.8
Alambo’20 [78]	Clinical notes	Linguistic, Tropical	Four binary ML2classifier	-	40/424/Adults	Precision, Recall, F-Measure	0.74, 0.68, 0.70
Tavasoli’21 [79]	EEG	13	RNN	Cold presser test	10/Adults	Accuracy	95.23%
Choi’21 [80]	PPG	-	CNN	-	120/Adults	AUC, Accuracy, *p*-value	0.659, 62.4%, <0.01
Winslow’22 [74]	ECG	Time/Frequency domain features	LR	Cold presser	41/Adults	Precision, Recall, F1 score	0.875, 0.785, 0.828
Baran’24 [75]	blood volume pulse and raw skin conductance	HRV, SC, PSS and Stress Vulnerability	Multiple Linear Regression	daily activities	67/old	f-static, *p*-values, *t*-values	-
Ayena’25 [76]	Accelerometers, optical sensors, EDA, ECG, EMG, respiration, temp	Movement intensity, HR, HRV, step count, skin conductance, perceived stress, STS	RF, MLM, XGBoost, CNN-LSTM, and LMMs	daily activities	688/Adults	Accuracy	37–91.67%

Sensors (Electrocardiogram (ECG), Galvanic Skin Response (GSR), Electromyogram (EMG), Photoplethysmography (PPG), Electroencephalogram (EEG), Sit-to-Stand (STS), Heart Rate (HR), Heart Rate Variability (HRV), Skin Conductance (SC), Perceived Stress (PSS)). Models: Multilevel Models (MLM); Linear Mixed-Effects Models (LMMs).

**Table 6:** Pain monitoring using wireless/remote-based approaches.

Paper	Technology Type	Machine Learning Technique
Stinson’13 [83]	Wi-Fi, web-based app, smart mobile device	–
Jacob’13 [87]	Smart mobile devices, local server, web-based app	–
Rajesh’13 [85]	WSN, soft-computing tools, smart mobile devices	–
Jibb’14 [84]	Soft-computing tools, smart mobile devices, web-based app	–
De La Vegra’14 [86]	Smart mobile devices, local servers, web-based app	–
Martínez’14 [89]	Web-based app, smart mobile devices	Knowledge-based system
Jiang’16 [90]	Wi-Fi with a cloud server	–
Jibb’17 [88]	Soft-computing tools, smart mobile devices, web-based app, computer vision algorithms, WSN	–
Atee’18 [82]	Web-based portal, smart mobile devices	Artificial intelligence algorithms
Yang’18 [91]	Mobile web application with cloud server	–
Rodriguez’20 [93]	IoT-based system with multiple communication protocols	–
Rastogi’20 [93]	Fog computing	–
Seles’20 [92]	GSM, Wi-Fi, cloud server, web-based app, smart mobile device	–
Ghoush’25 [94]	IoT, Multimodal Sentiment Analysis System, Cloud and Mobile Integration, Facial Recognition	CNN

**Table 7:** A comparison of publicly available databases focusing on pain in adults.

Dataset	Year	Subjects	Subject Type	Pain Type	Pain Levels	Modality Type
UNBC-McMaster Shoulder Pain [95]	2011	129	Self-Identified patients	Natural shoulder pain	0–16 (PSPI) & 0–10 (VAS)	Face videos
BioVid [46]	2013	90	Healthy volunteers	Simulated heat pain	1–4	Face videos
BP4D-Spontaneous [96]	2014	41	Healthy adults	Cold pressor task	0–2	Face videos
BP4D+ [97]	2016	140	Healthy adults	Cold pressor task	A–E	Face videos, Bio-potentials (heart rate, respiration rate, blood pressure, EDA)
EmoPainA [98]	2016	22	Chronic lower back pain patients	Physical exercises	Eight	Video, audio, motion capture, sEMG
MIntPAIN [40]	2017	20	Healthy volunteers	Simulated electrical pain	0–4	Face videos
SenseEmotion Database [69]	2017	45	Healthy adults	Heat stimulation	1–3	Bio-potential, facial images, audio signals (sEMG, ECG, SCL, respiration)
X-ITE PainA [99]	2019	134	Healthy adults	Heat and electrical stimulation	1–3	Face video, physiological signals (EDA, ECG, sEMG)
Delaware [52]	2020	276	Young adults	Physical pain	level 2, 5, 8, 10	Computer-Rendered Expressions, Posed Static Expressions
PainMonit [100]	2024	55	Healthy adults	Heat pain	1–4 level	BVP, EDA, skin temperature, ECG, EMG, IBI, HR, respiration

**Modality type** (ECG: electrocardiogram; EDA: electrodermal activity; sEMG: surface electromyography; SCL: skin conductance level).

## Data Availability

No new data were created or analyzed in this study. Data sharing is not applicable to this article.
